# Roles of *Arabidopsis *WRKY3 and WRKY4 Transcription Factors in Plant Responses to Pathogens

**DOI:** 10.1186/1471-2229-8-68

**Published:** 2008-06-20

**Authors:** Zhibing Lai, KM Vinod, Zuyu Zheng, Baofang Fan, Zhixiang Chen

**Affiliations:** 1 Department of Botany and Plant Pathology, 915 W. State Street, Purdue University, West Lafayette, IN 47907-2054, USA

## Abstract

**Background:**

Plant WRKY DNA-binding transcription factors are involved in plant responses to biotic and abiotic responses. It has been previously shown that *Arabidopsis WRKY3 *and *WRKY4*, which encode two structurally similar WRKY transcription factors, are induced by pathogen infection and salicylic acid (SA). However, the role of the two WRKY transcription factors in plant disease resistance has not been directly analyzed.

**Results:**

Both WRKY3 and WRKY4 are nuclear-localized and specifically recognize the TTGACC W-box sequences *in vitro*. Expression of *WRKY3 *and *WRKY4 *was induced rapidly by stress conditions generated by liquid infiltration or spraying. Stress-induced expression of *WRKY4 *was further elevated by pathogen infection and SA treatment. To determine directly their role in plant disease resistance, we have isolated T-DNA insertion mutants and generated transgenic overexpression lines for *WRKY3 *and *WRKY4*. Both the loss-of-function mutants and transgenic overexpression lines were examined for responses to the biotrophic bacterial pathogen *Pseudomonas syringae *and the necrotrophic fungal pathogen *Botrytis cinerea*. The *wrky3 *and *wrky4 *single and double mutants exhibited more severe disease symptoms and support higher fungal growth than wild-type plants after *Botrytis *infection. Although disruption of *WRKY3 *and *WRKY4 *did not have a major effect on plant response to *P. syringae*, overexpression of *WRKY4 *greatly enhanced plant susceptibility to the bacterial pathogen and suppressed pathogen-induced *PR1 *gene expression.

**Conclusion:**

The nuclear localization and sequence-specific DNA-binding activity support that WRKY3 and WRKY4 function as transcription factors. Functional analysis based on T-DNA insertion mutants and transgenic overexpression lines indicates that WRKY3 and WRKY4 have a positive role in plant resistance to necrotrophic pathogens and WRKY4 has a negative effect on plant resistance to biotrophic pathogens.

## Background

Upon pathogen infection, pathogen-associated molecular patterns (PAMPs) such as bacterial flagellin and lipopolysaccharides are recognized by plant receptors to activate PAMP-triggered immunity through a mitogen-activated protein kinase signaling cascade [1]. Gram-negative bacterial pathogens such as *Pseudomonas syringae *can deliver effector proteins to plant cells to interfere PAMP-triggered resistance to promote pathogen virulence. As a result, the remaining basal defense is usually insufficient to contain pathogens but can limit their growth in plant tissue. Through co-evolution, some effectors may be specifically recognized by plant resistance (R) proteins and activate strong effector-triggered immunity (ETI) [1]. R gene-activated ETI involves a complex defense program including production of reactive oxygen species (ROS) and salicylic acid (SA), rapid programmed cell death (hypersensitive responses, HR) and induction of a large number of host genes including pathogenesis-related (*PR*) genes [1]. In *Arabidopsis*, R gene- and SA-mediated defense mechanisms are effective against biotrophic pathogens that feed on living host tissue during the whole or part of their infection cycle [2,3].

Necrotrophic pathogens kill the host to extract nutrients. Many necrotrophic pathogens produce toxins, cell wall-degrading enzymes and ROS to promote disease and macerate plant tissue [4]. Plant defense mechanisms against necrotrophic pathogens have been analyzed relatively recently and appear to differ from those against biotrophic pathogens in important ways. First, gene-for-gene resistance is common to biotrophic pathogens but not to necrotrophic pathogens. Second, R gene-mediated HR is effective against biotrophic pathogens but does not deter and in some cases actually facilitate infection of necrotrophic pathogens [5]. Third, while SA is important for resistance to biotrophic pathogens, its role in defense against necrotrophic pathogens is limited, if any. In *Arabidopsis*, mutations that impair SA biosynthesis or signaling do not affect resistance to *Botrytis *[6,7]. Abolishing SA accumulation in transgenic *nahG *plants resulted in limited increase in susceptibility to *Botrytis *[6]. However, transgenic *nahG *plants have nonspecific phenotypes (i.g. reduced phytoalexin) independent of SA [8,9] and the enhanced susceptibility to *Botrytis *observed in transgenic *nahG *plants may not be caused by SA deficiency.

Although discovered relatively recently, WRKY transcription factors are becoming one of the best-characterized classes of plant transcription factors and are at the forefront of research on plant defense responses [10]. Pathogen infection or treatment with pathogen elicitors or SA induces rapid expression of plant WRKY genes. We have shown that in *Arabidopsis*, for example, expression of 49 out of 72 tested WRKY genes was differentially regulated after pathogen infection or SA treatment [11]. In addition, a large number of defense or defense-related genes, including well-studied *PR *genes and the regulatory *NPR1 *gene, contain W-box elements in their promoters that are specifically recognized by WRKY proteins and are necessary for their inducible expression [12-18]. More recent studies have provided direct evidence for the involvement of specific WRKY proteins in plant defense responses. For example, mutations of *Arabidopsis WRKY70 *enhance plant susceptibility to both biotrophic and necrotrophic pathogens including the bacterial pathogen *Erwinia carotovora *as well as fungal pathogens *Erysiphe cichoracearum *and *Botrytis *[19-21]. In addition, *wrky70 *mutants are compromised in both basal and R-gene (*RPP4*)-mediated resistance to the oomycete *Hyaloperonospora parasitica *[22]. *Arabidopsis wrky33 *mutants are highly susceptible to necrotrophic pathogens but respond normally to biotrophic pathogens [23]. These results indicate that WRKY33 plays an important and specific role in plant resistance to necrotrophic pathogens. Other WRKY proteins can function as negative regulators of plant disease resistance. For example, mutations of *Arabidopsis WRKY7*, *WRKY11 *and *WRKY17 *enhance plant resistance to virulent *P. syringae *strains [24-26] and mutations of *Arabidopsis WRKY25 *enhance tolerance to *P. syringae *[27]. The structurally related WRKY18, WRKY40 and WRKY60 function partially redundantly as negative regulators in plant resistance to *P. syringae *and *E. orontii *[28,29]. Their barley homologues HvWRKY1 and HvWRKY2 also function as suppressors of basal defense [29]. The diverse roles of WRKY proteins may reflect the complex signaling and transcriptional networks of plant defense that require tight regulation and fine-tuning.

We have previously shown that infection of an avirulent *P. syringae *strain or SA treatment induces *Arabidopsis WRKY3 *and *WRKY4*, which encode two structurally closely related WRKY proteins [11]. In the present study, we have shown that both WRKY3 and WRKY4 are nuclear-localized sequence-specific DNA-binding proteins. We have also shown that induced expression of *WRKY3 *and *WRKY4 *after pathogen infection or SA treatment was primarily due to plant stress caused by infiltration and spraying of pathogen suspension or SA solution. Both loss-of-function T-DNA insertion mutants and transgenic overexpression lines for *WRKY3 *and *WRKY4 *have been generated and examined for responses to the biotrophic bacterial pathogen *P. syringae *and the necrotrophic fungal pathogen *B. cinerea*. These studies strongly suggested that WRKY3 and WRKY4 play a positive role in plant resistance to necrotrophic pathogens but a negative role in resistance to biotrophic pathogens.

## Results

### Structures, DNA binding and subcellular localization

**Figure 1:**
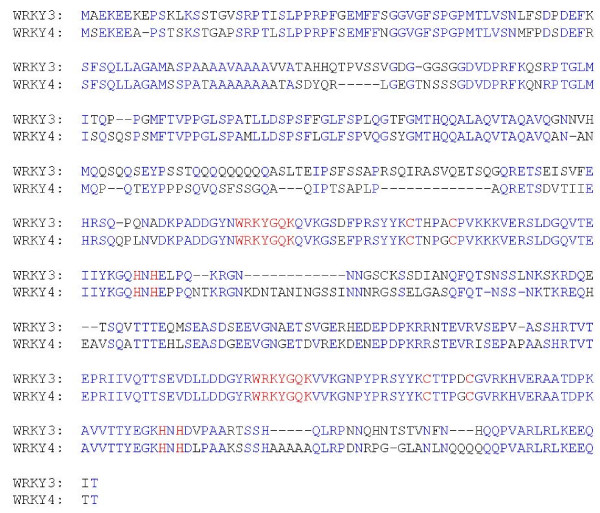
**WRKY3 and WRKY4 sequences and alignment**. Amino acid residues identical between the two proteins are blue. The highly conserved WRKYGQK sequences and the residues forming the C_2_H_2 _zinc-fingers are red.

Based on the number and structure of the WRKY zinc fingers, WRKY proteins are classified into three groups [30]. The first group contains two zinc-finger motifs while the second and third groups contain only one zinc-finger motif. The Cys_2_HisCys zinc finger in the third group of WRKY proteins is slightly different from the more common Cys_2_His_2 _zinc finger found in the first and second groups of WRKY proteins. WRKY3 and WRKY4 belong to group I WRKY proteins each with two Cys_2_His_2 _motifs (Figure 1). For group I WRKY proteins, the C-terminal WRKY zinc finger is responsible for sequence-specific DNA binding [31,32]. The NMR solution structure of the C-terminal DNA-binding WRKY zinc finger of the *Arabidopsis *WRKY4 protein consists of a four-stranded β-sheet, with a zinc binding pocket formed by the conserved Cys_2_His_2 _residues located at one end of the β-sheet to form a novel zinc and DNA binding structure [33]. Besides the conserved WRKY domains, WRKY3 and WRKY4 share high levels of similarity throughout their whole protein sequences (Figure 1).

**Figure 2:**
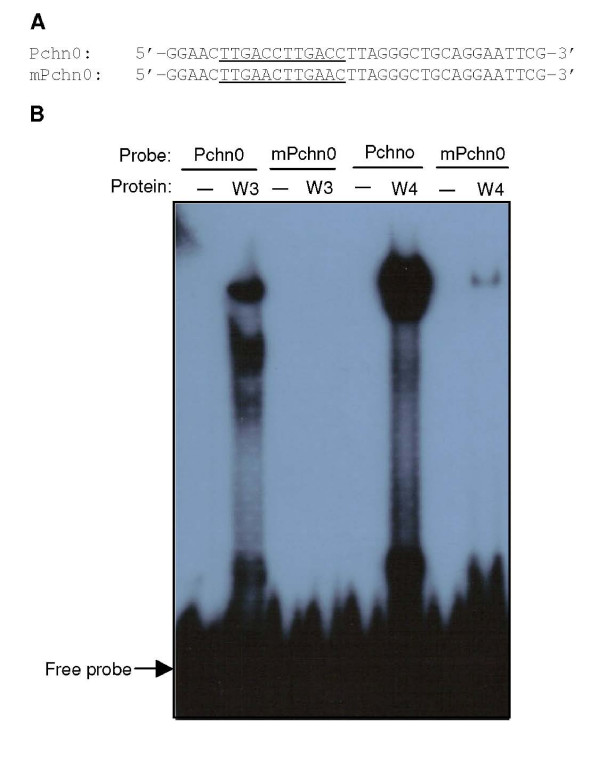
**DNA binding activity of WRKY3 and WRKY4**. **A**. Sequences of oligonucleotide probes. The Pchn0 probe contains two TTGACC W boxes, while in the mPchn0 probe, the TTGACC sequences are mutated to TTGAAC. The wild-type and mutated W-box sequences are underlined. **B**. Electrophoretic mobility shift assay (EMSA) of DNA binding of the WRKY3 and WRKY4 recombinant proteins.

Most characterized WRKY transcription factors recognize the TTGACC/T W-box sequences [12,15,34]. To examine the DNA-binding activity of WRKY3 and WRKY4, we expressed the genes in *E. coli*, purified the recombinant proteins, and assayed their binding to an oligonucleotide containing two direct TTGACC W-box repeats (Pchn0; Figure 2A) using electrophoretic mobility shifting assays (EMSA). Protein/DNA complexes with reduced mobility were detected when purified recombinant WRKY3 or WRKY4 protein was incubated with the Pchn0 probe (Figure 2B). Binding to a mutant probe (mPchn0) in which the TTGACC sequence was changed to TTGAAC was undetectable for WRKY3 and greatly reduced for WRKY4 (Figure 2B). Thus, binding of WRKY3 and WRKY4 to the TTGACC W-box sequence is highly specific.

**Figure 3:**
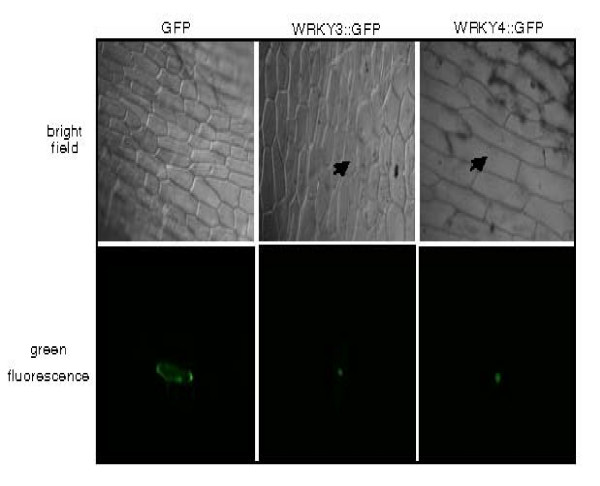
**Subcellular localization of WRKY3 and WRKY4**. WRKY3 and WRKY4 were fused to GFP to yield WRKY3::GFP and WRKY4::GFP, respectively; the chimeric proteins were localized to the nucleus of onion epidermal cells. GFP alone was detected in both the nucleus and the cytoplasm due to its small size. Bright-field image of the onion epidermal cells are shown in the top panels.

To determine the subcellular location of WRKY3 and WRKY4, we constructed GFP protein fusions of the two WRKY proteins at C terminal. The fusion constructs, driven by the *CaMV 35S *promoter, were directly bombarded into onion (*Allium cepa*) epidermal cells. The transiently expressed WRKY3-GFP and WRKY4-GFP fusion proteins were localized exclusively to the nucleus (Figure 3). By contrast, GFP was found in both the nucleus and cytoplasm (Figure 3).

### Expression

**Figure 4:**
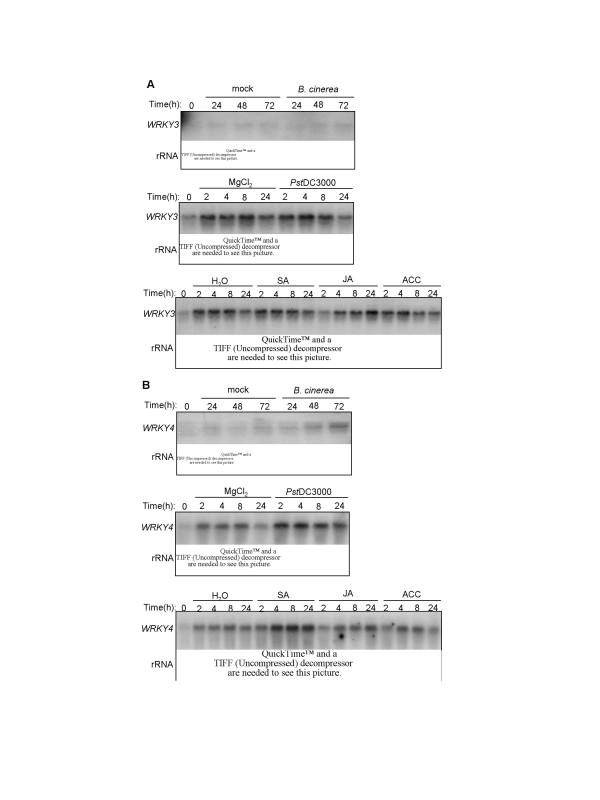
**Induced expression of *WRKY3 *and *WRKY4***. Five-week-old *Arabidopsis *plants (Col-0) were sprayed with either 1% maltose (mock) or *Botrytis*, infiltrated with 10 mM MgCl_2 _or *Pst*DC3000 (OD_600 _= 0.0001 in 10 mM MgCl_2_) or sprayed with H_2_O, SA (1 mM), methylJA (100 μM) or ACC (2 mM). The infiltrated or sprayed leaves were collected at indicated times after treatment for RNA isolation. RNA gel blot analysis was performed with a ^32^P-labeled probe for *WRKY3 *(**A**) or WRKY4 (**B**). The experiments were repeated two times with similar results.

To analyze the role of WRKY3 and WRKY4 in plant defense, we analyzed their expression after pathogen infection. Both mock (1% maltose only) and *Botrytis *infection resulted in a significant increase in the level of *WRKY3 *transcripts (Figure 4A). We also investigated its expression after infiltration with a control MgCl_2 _solution (mock inoculation) or a suspension of the virulent *P. syringae *pv. *tomato *strain DC3000 (*Pst*DC3000). As shown in Figure 4A, in plants infiltrated with MgCl_2 _or the bacterial suspension, the levels of *WRKY3 *transcripts were elevated at 2, 4, 8 and, to a lesser extent, 24 hours post infiltration (hpi) relative to that at 0 hpi (Figure 4A). *WRKY3 *transcripts were also rapidly elevated to similar levels after spraying with H_2_O, defense-inducing molecules SA, methyl jasmonic acid (JA) or 1-aminocyclopropane-1-carboxylic acid (ACC), the immediate precursor of ethylene (ET) biosynthesis (Figure 4B). Thus *WRKY3 *responded rapidly to stress conditions generated by liquid infiltration or spraying.

The expression of *WRKY4 *was responsive to both mock and *Botrytis *infection but at 48 and particularly 72 hpi, the levels of *WRKY4 *transcripts were higher in *Botrytis*-infected plants than those in mock-infected plants (Figure 4B). *WRKY4 *was also rapidly induced by infiltrated with either the MgCl_2 _solution or the bacterial suspension. However, in the three RNA blotting experiments performed, *WRKY4 *transcripts were consistently higher in pathogen-infiltrated plants than those in MgCl_2_-infiltrated plants (Figure 4B). Furthermore, the transcript levels of *WRKY4 *were consistently higher in SA-treated plants than those in H_2_O- JA- or ACC-sprayed plants. Thus, stress-induced expression of *WRKY4 *was further elevated by pathogen infection and SA treatment.

### Response of T-DNA insertion mutants to pathogens

**Figure 5:**
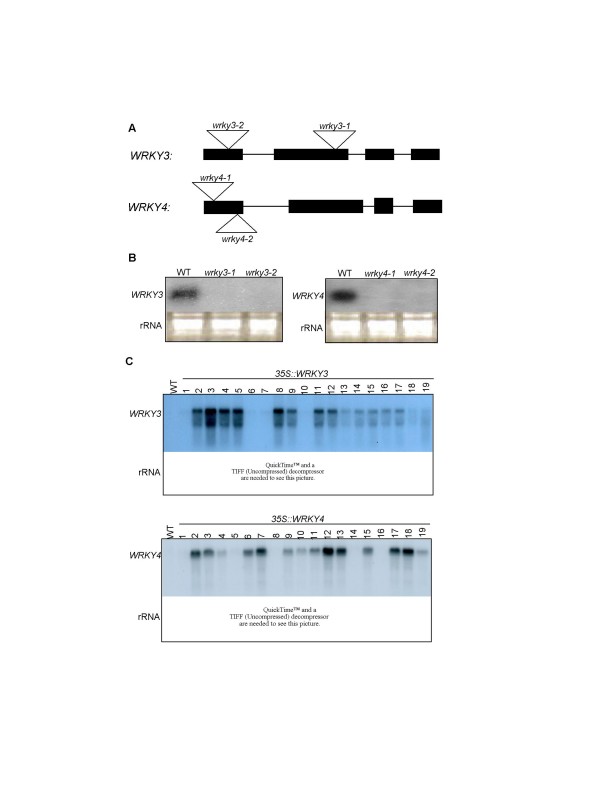
**Generation of T-DNA knockout and overexpression lines**. (**A**) Diagram of *WRKY3 *and *WRKY4 *and their T-DNA insertion mutants. **(B) **RNA gel blot analysis of *wrky3 *and *wrky4 *mutants. Wild-type and mutant plants were sprayed with SA (1 mM). The leaves were harvested 4 hours after treatment for total RNA isolation. After separation on the gels and blotting to nylon membranes, the blots were probed with corresponding gene-specific DNA fragments. **(C) ***WRKY3 *and *WRKY4 *expression in transgenic plants. RNA samples were prepared from leaves of 5-week-old wild-type (Col-0) and transgenic plants and probed with a *WRKY3*- or *WRKY4*-specific probe. Transgenic *WRKY3 *lines 3 and 8 and transgenic *WRKY4 *lines 7 and 13 contained a single T-DNA insertion in the genome and exhibited stable expression of their respective transgenes. Their F3 homozygous progeny plants were used in all the experiments in the study.

To determine the role of *WRKY3 *and *WRKY4 *directly, we identified two independent T-DNA insertion mutants for both *WRKY3 *and *WRKY4*. The *wrky3-1 *mutant (Salk_107019) contains a T-DNA insertion in the second exon while *wrky3-2 *(Salk_119051) contains a T-DNA insertion in the first exon of the *WRKY3 *gene (Figure 5A). The *wrky4-1 *(Salk_082016) and *wrky4-2 *(Salk_073118) mutants both contain a T-DNA insertion in the first exon of the *WRKY4 *gene (Figure 5A). Homozygous mutant plants were identified by PCR with *WRKY3*- or *WRKY4*-specific primers. Northern blotting analysis showed that *WRKY3 *or *WRKY4 *transcripts in the respective homozygous mutants were greatly reduced after SA treatment (Figure 5B). To determine possible functional redundancy, we also generated *wrky3-1/wrky4-1 *and *wrky3-2/wrky4-2 *double mutants through genetic crossing. The *wrky3 *and *wrky4 *single and double mutants show no difference in growth, development or morphology from wild-type plants. The *wrky3 *and *wrky4 *single and double mutants also responded normally to the virulent *Pst*DC3000 strain based on the growth of the bacterial pathogen. However, in two of the four experiments, *wrky4 *single mutant and *wrky3*/*wrky4 *double mutant plants developed significantly less chlorotic disease symptoms than wild-type plants after *Pst*DC3000 infection, suggesting that WRKY4 may play a negative role in plant tolerance to the bacterial pathogen.

**Figure 6:**
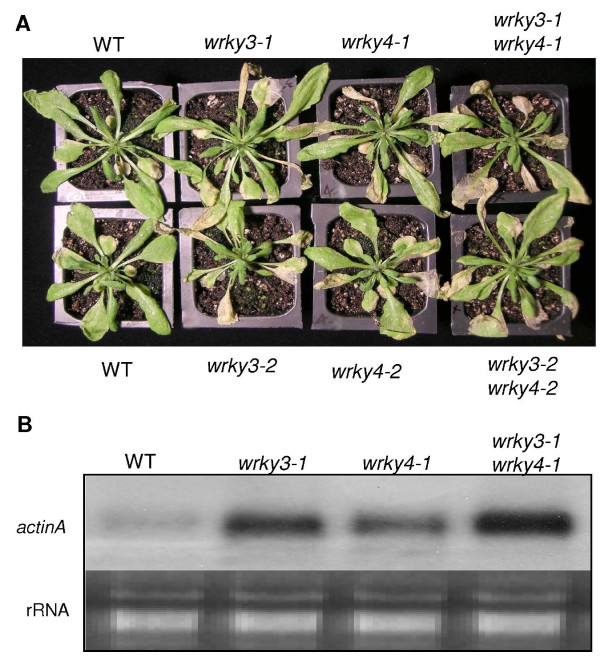
**Analysis of mutants to *Botrytis***. **(A) **Col-0 wild type (WT), *wrky3 *and *wrky4 *single and double mutant plants were inoculated by spraying spore suspension at a density of 5 × 10^5 ^spores/ml and kept at high humidity. The pictures of representative plants were taken 5 days after the inoculation. **(B) **The total RNA isolated from the plants 5 days after inoculation was probed with a *Botrytis *actinA gene probe to determine the biomass of the fungal pathogen on infected plants. The experiments were repeated three additional times with similar results.

To determine the role of WRKY3 and WRKY4 in plant resistance to a necrotrophic pathogen, we analyzed disease development caused by the infection of *B. cinerea *in the *wrky3 *and *wrky4 *mutant plants and compared it with that in wild-type plants. As shown in Figure 6A, the *wrky3 *and *wrky4 *single and double mutant plants developed more severe disease symptoms than wild-type plants. To quantify the fungal growth in these plants, we performed northern blotting of total RNA isolated from these plants using an actin gene from *B. cinerea *as probe. Like disease symptoms, accumulation of the transcript of the fungal gene was significantly elevated in the *wrky3 *and *wrky4 *single and double mutant plants (Figure 6B). These results suggest that WRKY3 and WRKY4 play a positive role in *Arabidopsis *resistance to the necrotrophic fungal pathogen.

### Responses of transgenic overexpression lines to pathogens

To further examine the roles of WRKY3 and WRKY4, we generated transgenic *Arabidopsis *plants that constitutively overexpress the WRKY genes. Constructs containing a full-length *WRKY3 *or *WRKY4 *cDNA driven by the *CaMV 35S *promoter were generated and transformed into *Arabidopsis *(Col-0 ecotype). Northern blotting identified several transgenic plants that contained elevated levels of *WRKY3 *transcripts even in the absence of SA treatment (Figure 3A). However, *WRKY3 *transcripts in the transgenic plants exhibited smear patterns on the RNA blot (Figure 5C), which could result from premature termination of transcription, alternative splicing and/or degradation of *WRKY3 *transcripts. Transgenic *WRKY4*-overexpressing lines were also identified, and *WRKY4 *transcripts in the overexpression lines were predominantly in a single band of expected size on the RNA blot (Figure 5C). The transgenic overexpression lines for WRKY3 and WRKY4 show no visible alteration in growth, development or morphology from wild-type plants.

**Figure 7:**
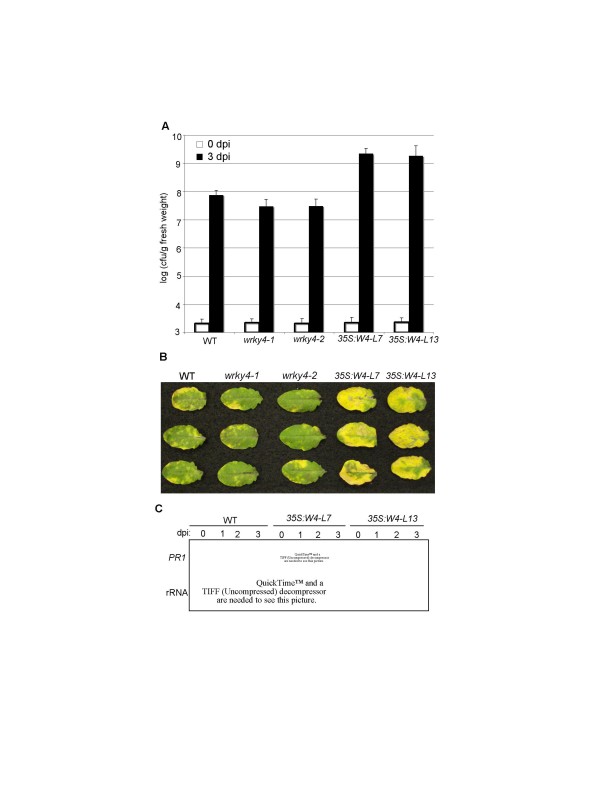
**Responses of *wrky4 *mutants and transgenic *WRKY4*-overexpressing plants to *Pst*DC3000**. **(A) **Altered bacterial growth in the WRKY mutants. Wild type (WT), *wrky4 *mutants and WRKY4-overexpressing lines 7 and 13 (*35S:W4-L7 *and *35S:W4-L13*) were infiltrated with a suspension of *Pst*DC3000 (OD_600 _= 0.0001 in 10 mM MgCl_2_). Samples were taken at 0 (open bars) or 3 days (closed bars) post inoculation (dpi) to determine the growth of the bacterial pathogen. The means and standard errors colony-forming units (cfu) were calculated from 10 plants for each treatment. **(B) **Disease symptom development. Pathogen inoculation of wild type, mutants and overexpression lines was performed as in **A**. Pictures of representative inoculated leaves taken at 4 dpi. **(C) **Pathogen-induced *PR1 *expression. Wild type and *WRKY4*-overexpressing plants were infiltrated with a suspension of *Pst*DC3000 (OD_600 _= 0.0001 in 10 mM MgCl_2_). Inoculated leaves were collected at indicated dpi for RNA isolation. RNA gel blot analysis was performed with ^32^P-labeled *PR1*. These experiments were repeated three times with similar results.

To determine possible changes of the overexpression lines in plant disease resistance, we first examined their responses to *B. cinerea *but found no significant difference in resistance to the fungal pathogen when compared to that of wild-type plants (data not shown). We then inoculated them with *Pst*DC3000 and monitored both bacterial growth and disease symptom development. We observed no significant difference between the *WRKY3*-overexpressing lines and wild-type plant in both disease symptom development and the growth of the bacterial pathogen. However, following inoculation with *Pst*DC3000, the transgenic *WRKY4 *overexpression lines displayed approximately 25–30 fold greater bacterial growth than wild-type plants (Figure 7A). The inoculated leaves of *WRKY4*-overexpressing plants also developed much more severe disease symptoms than those of wild-type plants after infection (Figure 7B). Thus overexpression of *WRKY4 *greatly enhanced plant susceptibility to the bacterial pathogen.

SA-mediated defense plays a critical role in plant defense against *P. syringae *[35,36]. SA-mediated defense mechanisms are associated with expression of *PR *genes including *PR1 *that is often used as a reliable molecular marker for SA-dependent systemic acquired resistance [35]. Since *WRKY4*-overexpressing plants had higher susceptibility than wild-type plants to *P. syringae*, we compared the transgenic plants with wild-type plants for pathogen-induced expression of *PR1*. These analyses revealed no significant *PR1 *transcript accumulation in buffer-treated wild type or *WRKY4*-overexpressing plants (Figure 7C). *PR1 *transcripts accumulated to high levels in wild-type plants at both 2 and 3 day after inoculation (dpi) (Figure 7C). In the *WRKY4*-overexpressing plants, however, accumulation of *PR1 *transcripts was substantially reduced at both 2 and 3 dpi when compared to that in wild-type plants (Figure 7C). The reduced *PR1 *expression in the *WRKY4*-overexpressing plants is consistent with reduced resistance of the overexpression plants to the bacterial pathogen.

## Discussion

*WRKY3 *and *WRKY4 *encode two structurally similar WRKY proteins (Figure 4) and their expression was both responsive to stress conditions (Figure 4). Stress-induced expression of *WRKY4 *but not *WRKY3 *was further enhanced by pathogen infection or SA treatment (Figure 4). Independent T-DNA insertion mutants for both *WRKY3 *and *WRKY4 *exhibited significantly more severe disease symptom and supported higher fungal growth than wild-type plants after infection by the necrotrophic fungal pathogen *B. cinerea *(Figure 6). Despite the similar structures of the two WRKY proteins, however, we observed no significant enhancement in the susceptibility to the fungal pathogen in the *wrky3*/*wrky4 *double mutant plants relative to the *wrky3 *and *wrky4 *single mutants (Figure 6). Thus there appeared to be little functional redundancy between the two structurally similar WRKY proteins in plant resistance to *B. cinerea*. We observed no major changes of the *wrky3 *and *wrky4 *single and double mutants to a virulent *P. syringae *strain (Figure 7), although a minor reduction in disease symptom development was observed in the *wrky4 *mutants in some of the experiments. On the other hand, overexpression of *WRKY4 *greatly reduced susceptibility to the bacterial pathogen based on strong enhancement in both bacterial growth and development of disease symptoms (Figure 7). No such enhancement in susceptibility to the bacterial pathogen was observed in transgenic *WRKY3*-overexpressing lines. The different phenotypes of the transgenic overexpression lines could be due to the distinct roles of the two WRKY proteins despite of their similar amino acid sequences. Alternatively, *WRKY3 *transcripts in the transgenic overexpression lines might not be full-length and, consequently, not functional for production of active WRKY3 proteins. Despite enhanced susceptibility of the *wrky3 *and *wrky4 *mutants to *B. cinerea*, we observed no increased resistance to the necrotrophic fungal pathogen in either *WRKY3*- or *WRKY4*-overexpressing lines. This might be due to the fact that the Col-0 wild-type accession, in which the transgenic overexpression lines were produced, is already quite resistant to *B. cinerea *(Figure 6). These results collectively indicate that WRKY3 and WRKY4 play a positive role in plant resistance to *Botrytis *while overexpression of, at least, WRKY4 has a strong negative effect on plant resistance to *P. syringae*.

SA-mediated signaling pathways are important for disease resistance to *P. syringae*, a biotrophic or more precisely hemitrophic pathogen that rely on living plant tissue during its early stages of infection [35,36]. On the other hand, JA/ET-mediated defense is important for plant resistance necrotrophic pathogens such as *Botrytis *[35]. SA and ET/JA signaling pathways are mutually antagonistic [36]. As a result, mutations of JA signaling regulators such as COI1 and MPK4 can enhance SA accumulation and signaling in pathogen-infected plants, resulting in elevated resistance to biotrophic and hemibiotrophic pathogens. Likewise, blocking SA accumulation can promote JA-regulated genes in *Arabidopsis *plants after infection by *P. syringae *[37]. Overexpression of WRKY4 resulted in enhanced plant susceptibility to *P. syringae *and reduced expression of SA-regulated *PR1 *gene expression (Figure 7). Thus WRKY4 has a negative effect on SA-mediated signaling pathways. On the other hand, WRKY3 and WRKY4 play a positive role in JA/ET-regulated resistance to necrotrophic pathogens based on the susceptible phenotypes to *Botrytis *(Figure 6). These results strongly suggest that WRKY4 and perhaps WRKY3 as well regulate crosstalk between SA- and JA/ET-mediated signaling pathways and, as a result, play opposite roles in resistance to the two different types of microbial pathogens.

A number of early studies of WRKY transcription factors have suggested their roles in SA-regulated defense responses. Many plant WRKY genes are induced by biotrophic patogens including avirulent *P. syringae *in *Arabidopsis *and tobacco mosaic virus (TMV) in tobacco that are known to induce SA-dependent SAR [11,34]. A reported microarray experiment has revealed that the promoters of genes co-induced with PR1 during the development of SAR in *Arabidopsis *are enriched in W boxes, suggesting a critical role of WRKY transcription factors in induction of SAR-associated genes [38]. We have previously shown that the W box sequences in the promoter of the *NPR1 *regulatory gene required for SA signaling are important for its expression [15]. Intriguingly, functional analysis of individual WRKY genes has so far revealed a different picture about the roles of WRKY proteins in SA-mediated defense against biotrophic pathogens. *Arabidopsis wrky7*, *wrky11 *and *wrky17 *mutants are more susceptible to virulent *P. syringae *strains than wild-type plants [24-26] and mutations of *Arabidopsis WRKY25 *enhance tolerance to *P. syringae *[27]. *Arabidopsis *WRKY18, WRKY40 and WRKY60 function partially redundantly as negative regulators in plant resistance to *P. syringae *and *E. orontii *[28,29]. Their barley homologues HvWRKY1 and HvWRKY2 also suppress basal defense [29]. We have also previously shown that *overexpression *of *Arabidopsis WRKY25 *and *WRKY33 *enhances plant susceptibility to *P. syringae *and suppresses pathogen-induced *PR *gene expression [23,27]. Thus, these characterized WRKY proteins function as negative regulators of SA-mediated defense. Interestingly, some of these WRKY proteins such as WRKY4, WRKY33 and redundant WRKY18, WRKY40 and WRKY60 play a positive role in plant resistance to necrotrophic pathogens [23,28]. One exception is *Arabidopsis *WRKY70, which is shown to regulate the crosstalk by activating SA signaling but suppressing JA-mediated signaling [19,20]. Intriguingly, the *wrky70 *mutants, which are expected to be compromised in SA signaling and active in JA signaling, responded normally to *P. syringae *but exhibited enhanced susceptibility to *Botrytis *[19-21]. Therefore, the roles of WRKY70 in plant defense signaling pathways might be complicated.

## Conclusion

Crosstalk between defense signaling pathways is an important mechanism for regulating defense mechanisms against different types of microbial pathogens. Although genes capable of regulating the crosstalk have been reported, the underlying mechanisms have not been clearly understood. Identification of a number of WRKY transcription factors that affect plant resistance in opposite ways to different types of microbial pathogens suggest that the regulation of the crosstalk between these defense signaling pathways occurs at the transcription level. For example, some of these WRKY transcription factors may regulate the crosstalk by activating expression of JA/ET-regulated genes but repressing SA-regulated genes. Such direct and opposite roles in regulation of gene expression would require these WRKY transcription factors acting as transcriptional activators or repressors in a gene-specific manner. Alternatively, the WRKY transcription factors regulate the crosstalk by activating expression of genes associated with JA/ET-mediated signaling pathways, including some encoding transcriptional repressor that suppress SA-regulated gene expression. Further studies on the transcriptional activation/repression activities and direct transcriptional targets of the WRKY proteins could generate important insights into the molecular network of complex plant defense responses to different types of microbial pathogens.

## Methods

### Materials

^32^P-dATP (>3000 Ci/mmol) was obtained from DuPont-New England Nuclear; other common chemicals were purchased from Sigma. *Arabidopsis thaliana *plants were grown in a growth chamber at 24°C under 100 μE·m^-2^·sec^-1 ^light with 12-hr-light/12-hr-dark photoperiod. *Pst*DC3000 were maintained on King's B medium containing 100 μg/ml of rifampicin and 50 μg/ml kanamycin. The conidiospores of *B. cineria *isolate B5-10 were collected from 10 days old mycelium growing on V8-agar medium and suspended in 1% maltose for inoculation as previously described [23].

### Recombinant protein and DNA-binding

Full-length cDNAs for *WRKY3 *and *WRKY4 *were isolated from an *Arabidopsis *cDNA library constructed from SA-treated *Arabidopsis *plants as previously described [39]. For production of recombinant WRKY3 and WRKY4 proteins, full-length *WRKY3 *and *WRKY4 *coding sequences were amplified by PCR using gene-specific primers (5'-ATCGAATTCATGGCGGAGAAGGAAGAAAAAG-3' and 5'-ATCCTCGAGCTAAGCCATGGTGATTTGCTCTTCTTTAAGCCT-3' for *WRKY3 *and 5'-ATCGAATTCATGTCGGAAAAGGAAGAAGCTC-3' and 5'-ATCCTCGAGCTAAGCCATGGTTGTTTGCTCTTCTTTAAGCCT-3' for *WRKY4*). The amplified PCR fragments were digested with *EcoRI *and *XhoI *and cloned into the same sites of the pET-32a *E. coli *expression vector. Preparation of recombinant proteins and DNA-binding assays were performed as previously described [15].

### Subcellular localization

Full-length *WRKY3 *and *WRKY4 *coding sequences were amplified by PCR with the same gene-specific primers used for generating expression constructs in *E. coli *as described above. The amplified PCR fragments were digested with *EcoRI *and *NcoI *and cloned into the same sites of a GFP fusion expression vector as previously described [23,25]. Onion epidermal cell layers were peeled and placed inside up on the MS plates. Plasmid DNAs of appropriate fusion genes (0.5 μg) were introduced to the onion cells using a pneumatic particle gun (PDS 1000, Du Pont). The condition of bombardment was vacuum of 28 inch Hg, helium pressure of 1100 or 1300 psi, and 6 cm of target distance using 1.1 μm of tungsten microcarriers. After bombardment, tissues were incubated on the MS plates for 24 h at 22°C. Samples were observed directly or transferred to glass slides.

### Identification of the wrky3 and wrky4 T-DNA insertion mutants

The *wrky3-1 *mutant (Salk_107019) contains a T-DNA insertion in the second exon while *wrky3-2 *(Salk_119051) contains a T-DNAinsertion in the first exon of the *WRKY3 *gene (Figure 5A). The *wrky4-1 *(Salk_082016) and *wrky4-2 *(Salk_073118) mutants both contain a T-DNA insertion in the first exon of the *WRKY4 *gene. T-DNA insertions were confirmed by PCR using a combination of a T-DNA border primer (5'-GCTTGCTGCAACTCTCTCAG-3') and a gene specific primer (5'-GCTTCATTGACTGAGATTCCATC-3', 5'-CCCGGTGGTTGAGTTATCAT-3', 5'-TCATCGGAATCAGGGAACAT-3' and 5'-TCATCGGAATCAGGGAACAT-3' for *wrky3-1*, *wrky3-2*, *wrky4-1*, and *wrky4-2*, respectively). The nature and location of the T-DNA insertion was confirmed by sequencing the PCR products. Homozygous T-DNA mutant plants were identified by PCR using primers corresponding to sequences flanking the T-DNA insertion sites (the above four gene-specific primers paired with 5'-ATTCCCAACCTCCTCGCTAT-3' for *wrky3-1*, 5'-GAGAAACACGACACGAATTTTG-3' for *wrky3-2*, 5'-AAACACGACACGGATTCACA-3' for *wrky4-1 *and *wrky4-2*, respectively). To remove additional T-DNA loci or mutations from the mutants, we backcrossed them to wild-type plants and identified plants homozygous for the T-DNA insertion.

### Generation of transgenic WRKY3 and WRKY4 overexpression plants

Full-length *WRKY3 *and *WRKY4 *cDNAs were excised from the cloning vectors using BamHI and XhoI and subcloned into the *BamHI *and *SalI *sites behind the *CaMV 35S *promoter in pOCA30 [23,25]. *Arabidopsis *transformation was performed by the flora-dip procedure [40] and transformants were identified by screening for kanamycin resistance. From the transformants, those with a single copy of T-DNA insertion (based on the 3:1 segregation of antibiotic resistance in T2 progeny) were isolated and homozygote transgenic plants were further identified in the T3 generation based on the segregation in antibiotic resistance.

### RNA gel blotting

For RNA gel blot analysis, total RNA was extracted with Trizol reagent (Invitrogen) from leaf tissue, separated on 1.2% agarose-formaldehyde gels and blotted to nylon membranes according to standard procedures. Blots were hybridized with – ^32^P-dATP-labeled gene-specific probes. Hybridization in PerfectHyb™ Plus hybridization buffer (Sigma) at 68°C and subsequent membrane washing were performed as previously described. Full-length cDNAs were used as probes in Northern blotting for detecting *WRKY3 *and *WRKY4 *transcripts. *Arabidopsis PR1 *gene probe was generated from a *PR1 *DNA fragment amplified by PCR using two *PR1*-specific primers (5'-TTCTTCCCTCGAAAGCTCAA-3' and 5'-CGTTCACATAATTCCCACGA-3'). The *B. cinerea ActinA *gene probe [41] was amplified from the *B. cinerea *genomic DNA and by PCR using primers 5'-ACTCATATGTTGGAGATGAAGCGCA-3' and 5'-TGTTACCATACAAATCCTTACGGACA-3'.

### Pathogen inoculation and disease development

For disease resistance to *P. syringae*, three mature leaves of each 5-weeks old plant were infiltrated with a virulent strain (OD_600 _= 0.0001 in 10 mM MgCl_2_). The bacterial titers were determined immediately after infiltration or after 3 days post-inoculation for bacteria growth analysis. For disease resistance to *B. cineria*, the fungal spores (5 × 10^5 ^spores/ml) were sprayed on 35 day-old plants evenly. The plants were covered with transparent plastic dome to maintain high humidity and disease development was evaluated 5 days later.

## Authors' contributions

ZL carried out expression analysis of the WRKY genes and analysis of the mutants and overexpression lines. KMV carried out DNA binding assays, determination of subcellular localization, isolation, generation and characterization of the mutants and transgenic overexpression lines. ZZ carried out *Botrytis *tests. BF carried out isolation of the cDNA clones. ZC conceived of the study, participated in the design and helped to draft and edit the manuscript. All authors read and approved the final manuscript.

## References

[B1] Jones JD, Dangl JL (2006). The plant immune system. Nature.

[B2] Dong X (1998). SA, JA, ethylene, and disease resistance in plants. Curr Opin Plant Biol.

[B3] McDowell JM, Cuzick A, Can C, Beynon J, Dangl JL, Holub EB (2000). Downy mildew (*Peronospora parasitica*) resistance genes in *Arabidopsis* vary in functional requirements for NDR1, EDS1, NPR1 and salicylic acid accumulation. Plant J.

[B4] Prins TW, Tudzynski P, Tiedemann AV, Tudzynski B, eds (2000). Infection strategies of Botrytis cinerea and related necrotrophic pathogens.

[B5] Govrin EM, Levine A (2000). The hypersensitive response facilitates plant infection by the necrotrophic pathogen *Botrytis cinerea*. Curr Biol.

[B6] Ferrari S, Plotnikova JM, De Lorenzo G, Ausubel FM (2003). *Arabidopsis* local resistance to *Botrytis cinerea* involves salicylic acid and camalexin and requires EDS4 and PAD2, but not SID2, EDS5 or PAD4. Plant J.

[B7] Veronese P, Nakagami H, Bluhm B, Abuqamar S, Chen X, Salmeron J, Dietrich RA, Hirt H, Mengiste T (2006). The membrane-anchored BOTRYTIS-INDUCED KINASE1 plays distinct roles in *Arabidopsis* resistance to necrotrophic and biotrophic pathogens. Plant Cell.

[B8] Heck S, Grau T, Buchala A, Metraux JP, Nawrath C (2003). Genetic evidence that expression of NahG modifies defence pathways independent of salicylic acid biosynthesis in the *Arabidopsis-Pseudomonas syringae* pv. *tomato* interaction. Plant J.

[B9] van Wees SC, Glazebrook J (2003). Loss of non-host resistance of *Arabidopsis NahG* to *Pseudomonas syringae* pv. *phaseolicola* is due to degradation products of salicylic acid. Plant J.

[B10] Eulgem T, Somssich IE (2007). Networks of WRKY transcription factors in defense signaling. Curr Opin Plant Biol.

[B11] Dong J, Chen C, Chen Z (2003). Expression profile of the *Arabidopsis* WRKY gene superfamily during plant defense response. Plant Mol Biol.

[B12] Rushton PJ, Torres JT, Parniske M, Wernert P, Hahlbrock K, Somssich IE (1996). Interaction of elicitor-induced DNA-binding proteins with elicitor response elements in the promoters of parsley PR1 genes. Embo J.

[B13] Yang P, Wang Z, Fan B, Chen C, Chen Z (1999). A pathogen- and salicylic acid-induced WRKY DNA-binding activity recognizes the elicitor response element of the tobacco class I chitinase gene promoter. Plant J.

[B14] Willmott RL, Rushton PJ, Hooley R, Lazarus CM (1998). DNase1 footprints suggest the involvement of at least three types of transcription factors in the regulation of alpha-Amy2/A by gibberellin. Plant Mol Biol.

[B15] Yu D, Chen C, Chen Z (2001). Evidence for an important role of WRKY DNA binding proteins in the regulation of NPR1 gene expression. Plant Cell.

[B16] Rocher A, Dumas C, Cock JM (2005). A W-box is required for full expression of the SA-responsive gene SFR2. Gene.

[B17] Turck F, Zhou A, Somssich IE (2004). Stimulus-dependent, promoter-specific binding of transcription factor WRKY1 to Its native promoter and the defense-related gene PcPR1-1 in Parsley. Plant Cell.

[B18] Yamamoto S, Nakano T, Suzuki K, Shinshi H (2004). Elicitor-induced activation of transcription via W box-related cis-acting elements from a basic chitinase gene by WRKY transcription factors in tobacco. Biochim Biophys Acta.

[B19] Li J, Brader G, Palva ET (2004). The WRKY70 transcription factor: a node of convergence for jasmonate-mediated and salicylate-mediated signals in plant defense. Plant Cell.

[B20] Li J, Brader G, Kariola T, Palva ET (2006). WRKY70 modulates the selection of signaling pathways in plant defense. Plant J.

[B21] AbuQamar S, Chen X, Dhawan R, Bluhm B, Salmeron J, Lam S, Dietrich RA, Mengiste T (2006). Expression profiling and mutant analysis reveals complex regulatory networks involved in *Arabidopsis* response to *Botrytis* infection. Plant J.

[B22] Knoth C, Ringler J, Dangl JL, Eulgem T (2007). *Arabidopsis* WRKY70 is required for full RPP4-mediated disease resistance and basal defense against *Hyaloperonospora parasitica*. Mol Plant Microbe Interact.

[B23] Zheng Z, Qamar SA, Chen Z, Mengiste T (2006). *Arabidopsis* WRKY33 transcription factor is required for resistance to necrotrophic fungal pathogens. Plant J.

[B24] Park CY, Lee JH, Yoo JH, Moon BC, Choi MS, Kang YH, Lee SM, Kim HS, Kang KY, Chung WS (2005). WRKY group IId transcription factors interact with calmodulin. FEBS Lett.

[B25] Kim KC, Fan B, Chen Z (2006). Pathogen-Induced *Arabidopsis* WRKY7 Is a Transcriptional Repressor and Enhances Plant Susceptibility to *Pseudomonas syringae*. Plant Physiol.

[B26] Journot-Catalino N, Somssich IE, Roby D, Kroj T (2006). The Transcription Factors WRKY11 and WRKY17 Act as Negative Regulators of Basal Resistance in *Arabidopsis thaliana*. Plant Cell.

[B27] Zheng Z, Mosher SL, Fan B, Klessig DF, Chen Z (2007). Functional analysis of *Arabidopsis* WRKY25 transcription factors in plant defense against *Pseudomonas syringae*. BMC Plant Biol.

[B28] Xu X, Chen C, Fan B, Chen Z (2006). Physical and Functional Interactions between Pathogen-Induced *Arabidopsis* WRKY18, WRKY40, and WRKY60 Transcription Factors. Plant Cell.

[B29] Shen QH, Saijo Y, Mauch S, Biskup C, Bieri S, Keller B, Seki H, Ulker B, Somssich IE, Schulze-Lefert P (2007). Nuclear activity of MLA immune receptors links isolate-specific and basal disease-resistance responses. Science.

[B30] Eulgem T, Rushton PJ, Robatzek S, Somssich IE (2000). The WRKY superfamily of plant transcription factors. Trends Plant Sci.

[B31] de Pater S, Greco V, Pham K, Memelink J, Kijne J (1996). Characterization of a zinc-dependent transcriptional activator from *Arabidopsis*. Nucleic Acids Res.

[B32] Ishiguro S, Nakamura K (1994). Characterization of a cDNA encoding a novel DNA-binding protein, SPF1, that recognizes SP8 sequences in the 5' upstream regions of genes coding for sporamin and beta-amylase from sweet potato. Mol Gen Genet.

[B33] Yamasaki K, Kigawa T, Inoue M, Tateno M, Yamasaki T, Yabuki T, Aoki M, Seki E, Matsuda T, Tomo Y (2005). Solution Structure of an *Arabidopsis* WRKY DNA Binding Domain. Plant Cell.

[B34] Chen C, Chen Z (2000). Isolation and characterization of two pathogen- and salicylic acid-induced genes encoding WRKY DNA-binding proteins from tobacco. Plant Mol Biol.

[B35] Glazebrook J (2005). Contrasting Mechanisms of Defense Against Biotrophic and Necrotrophic Pathogens. Annu Rev Phytopathol.

[B36] Kunkel BN, Brooks DM (2002). Cross talk between signaling pathways in pathogen defense. Curr Opin Plant Biol.

[B37] Spoel SH, Koornneef A, Claessens SM, Korzelius JP, Van Pelt JA, Mueller MJ, Buchala AJ, Metraux JP, Brown R, Kazan K (2003). NPR1 modulates cross-talk between salicylate- and jasmonate-dependent defense pathways through a novel function in the cytosol. Plant Cell.

[B38] Maleck K, Levine A, Eulgem T, Morgan A, Schmid J, Lawton KA, Dangl JL, Dietrich RA (2000). The transcriptome of *Arabidopsis thaliana* during systemic acquired resistance. Nat Genet.

[B39] Chen C, Chen Z (2002). Potentiation of developmentally regulated plant defense response by AtWRKY18, a pathogen-induced *Arabidopsis* transcription factor. Plant Physiol.

[B40] Clough SJ, Bent AF (1998). Floral dip: a simplified method for *Agrobacterium*-mediated transformation of *Arabidopsis thaliana*. Plant J.

[B41] Benito EP, ten Have A, van 't Klooster JW, van Kan JAL (1998). Fungal and plant gene expression during synchronized infection of tomato leaves by *Botrytis cinerea*. European Journal of Plant Pathology.

